# Ticagrelor versus clopidogrel in STEMI post-PCI: A mixed-design meta-analysis of efficacy and safety

**DOI:** 10.1097/MD.0000000000049923

**Published:** 2026-07-24

**Authors:** Mohammad Maroof Shahid, Muhammad Hamza Sajjad, Sanket Kumar Pankajbhai Patel, Moosa Feroze Tarar, Malik Abdullah Rasheed, Muhammad Soban Jaffar, Muhammad Ali Rana, Mohammad Hamza Abbas, Abdul Basit Rasheed, Maira Shahid, Mirza Muhammad Hadeed Khawar, Elham Shenawa

**Affiliations:** a Department of Cardiology, Services Institute of Medical Sciences, Lahore, Pakistan; b Department of Cardiology, Our Lady of Lourdes Hospital, Drogheda, Ireland; c Department of Cardiology, Tianjin Medical University, Tianjin, China; d Department of Cardiology, King Edward Medical University, Lahore, Pakistan; e Department of Internal Medicine, Pakistan Kidney and Liver Institute and Research Center, Lahore, Pakistan; f Department of Internal Medicine, Faisalabad Medical University, Faisalabad, Pakistan; g Department of Cardiology, Balkh University Faculty of Medicine, Balkh, Afghanistan.

**Keywords:** bleeding risk, clopidogrel, dual antiplatelet therapy, major adverse cardiovascular events, primary percutaneous coronary intervention, ST-segment elevation myocardial infarction, stent thrombosis, ticagrelor

## Abstract

**Background:**

ST-segment elevation myocardial infarction (STEMI) requires primary percutaneous coronary intervention (PCI) and dual antiplatelet therapy with aspirin and a purinergic receptor Y12 inhibitor to reduce thrombotic risks. Ticagrelor, a potent purinergic receptor Y12 inhibitor, offers faster and stronger platelet inhibition than clopidogrel, but evidence on its efficacy and safety in STEMI patients post-PCI is conflicting. This study aims to compare the efficacy and safety of ticagrelor versus clopidogrel in STEMI patients undergoing primary PCI.

**Methods:**

This systematic review and meta-analysis, adhering to Preferred Reporting Items for Systematic Reviews and Meta-Analyses 2020 guidelines, searched PubMed, Embase, ScienceDirect, and ClinicalTrials.gov up to July 2025. Included were randomized controlled trials and observational studies comparing ticagrelor to clopidogrel in adult STEMI patients post-PCI, reporting cardiovascular outcomes. Data were pooled using odds ratios (ORs) with 95% confidence intervals (CIs) via random-effects models.

**Results:**

Eight studies (5 randomized controlled trials, 3 observational; n = 31,729) showed ticagrelor significantly reduced all-cause mortality (OR = 0.64, 95% CI = 0.47–0.88; *P* = .006), cardiovascular mortality (OR = 0.68, 95% CI = 0.60–0.93; *P* = .01), major adverse cardiovascular events (OR = 0.71, 95% CI = 0.60–0.84; *P* = .01), myocardial infarction (OR = 0.71, 95% CI = 0.61–0.83; *P* < .00001), stent thrombosis (OR = 0.66, 95% CI = 0.55–0.79; *P* < .00001), and major bleeding (OR = 0.87, 95% CI = 0.78–0.97; *P* = .01), but increased target vessel revascularization (OR = 1.30, 95% CI = 1.05–1.61; *P* = .02). No significant difference was observed in stroke risk (OR = 1.16, 95% CI = 0.89–1.52; *P* = .28).

**Conclusion:**

Ticagrelor outperforms clopidogrel in STEMI post-PCI, reducing key adverse outcomes, though higher target vessel revascularization risk warrants caution.

## 1. Introduction

ST-segment elevation myocardial infarction (STEMI) represents the most severe form of acute coronary syndrome, requiring immediate reperfusion therapy to minimize myocardial necrosis and improve clinical outcomes.^[1]^ Primary percutaneous coronary intervention (PCI) has emerged as the preferred reperfusion strategy for STEMI patients when performed on time.^[2,3]^ Dual antiplatelet therapy, combining aspirin with a purinergic receptor Y12 (P2Y12) inhibitor, constitutes a cornerstone of pharmacological management during and after primary PCI to prevent stent thrombosis and reduce ischemic complications.^[4,5]^

The landscape of antiplatelet therapy has evolved significantly with the introduction of newer, more potent P2Y12 inhibitors. While clopidogrel was traditionally the standard P2Y12 inhibitor, ticagrelor has emerged as a more potent alternative, offering faster onset of action, greater platelet inhibition, and reversible binding to the P2Y12 receptor. The landmark PLATO trial demonstrated the superiority of ticagrelor over clopidogrel in reducing cardiovascular death, myocardial infarction (MI), and stroke in patients with acute coronary syndromes. However, the subgroup analysis of STEMI patients in PLATO showed only a numerical trend toward benefit without reaching statistical significance for the primary endpoint.^[6,7]^

Despite guideline recommendations favoring ticagrelor over clopidogrel in STEMI patients, several critical knowledge gaps persist that warrant systematic evaluation through meta-analysis. The individual studies comparing ticagrelor and clopidogrel specifically in STEMI patients undergoing primary PCI have yielded conflicting results. While some studies demonstrate reduced mortality and ischemic events with ticagrelor, others report no significant difference in major adverse cardiovascular events (MACE).^[8]^ The TREAT trial, focusing on STEMI patients receiving fibrinolytic therapy, found no significant difference in efficacy outcomes between ticagrelor and clopidogrel at 12 months.^[9]^ Substantial heterogeneity exists in patient populations across studies, with varying ages, comorbidities, and risk profiles potentially influencing treatment effects. Elderly patients (≥75 years) represent a particularly vulnerable population where the balance between ischemic benefit and bleeding risk becomes critical. The Bremen STEMI Registry demonstrated efficacy benefits of ticagrelor in elderly patients, while other studies raised concerns about increased bleeding risk in this population.^[10]^ Real-world evidence has challenged the findings of randomized controlled trials (RCTs), with several observational studies reporting no significant difference in MACE between ticagrelor and clopidogrel. These discrepancies between trial and real-world data suggest potential selection bias in randomized trials and highlight the need for comprehensive analysis across diverse study designs.^[11]^

The bleeding risk profile of ticagrelor remains a matter of debate, with some randomized trials and observational analyses reporting comparable safety to clopidogrel – including no significant difference in major bleeding among STEMI patients with cardiogenic shock undergoing primary PCI or in those managed with a pharmaco-invasive strategy,^[12]^ while others have reported increased bleeding events, such as higher perioperative bleeding in STEMI patients undergoing early coronary artery bypass grafting after ticagrelor preloading.^[13]^ This uncertainty is particularly relevant in STEMI populations, where baseline bleeding risk is amplified by the acute presentation, hemodynamic instability at admission, and the frequent need for multiple antithrombotic agents as part of contemporary reperfusion strategies.

Given these knowledge gaps and the clinical importance of optimizing antiplatelet therapy in STEMI patients undergoing primary PCI, a comprehensive meta-analysis is needed to provide definitive evidence on the comparative efficacy and safety of ticagrelor versus clopidogrel. This systematic evaluation will synthesize data from RCTs and observational studies to determine whether ticagrelor offers superior clinical outcomes compared to clopidogrel in this high-risk population, while accounting for potential sources of heterogeneity, reconciling discrepancies between trial and real-world data, and providing evidence-based guidance for clinical practice.

## 2. Materials and methods

This systematic review and meta-analysis were conducted per the Preferred Reporting Items for Systematic Reviews and Meta-Analyses 2020 guidelines.^[14]^ The PROSPERO protocol has been registered (ID: CRD420251144140). Ethical approval was not required for this study as it is a systematic review and meta-analysis, and it does not involve the collection of primary data from human participants.

### 2.1. Eligibility criteria

#### 2.1.1. Inclusion criteria

The inclusion criteria for this systematic review and meta-analysis included studies involving adult patients aged 18 years or older who were diagnosed with STEMI and underwent primary PCI. Eligible studies compared ticagrelor with clopidogrel as part of dual antiplatelet therapy, with or without concomitant aspirin. Studies were required to report at least one of the following outcomes with a minimum follow-up of 30 days, prioritizing 1-year data when available: MI, stent thrombosis, stroke, target vessel revascularization (TVR), all-cause mortality, cardiovascular mortality, MACE, or major bleeding, as defined by individual studies. Only RCTs and observational cohort studies published in English with full-text availability were included, with no restrictions on publication year.

#### 2.1.2. Exclusion criteria

The exclusion criteria encompassed studies involving patients with non-STEMI or non-STEMI acute coronary syndromes, those not undergoing primary PCI, and studies that did not directly compare ticagrelor with clopidogrel. In addition, noncomparative studies, case reports, editorials, expert opinions, and duplicates were excluded.

### 2.2. Information sources and search strategy

The search strategy for this meta-analysis was designed to retrieve studies from PubMed, Embase, ScienceDirect, and ClinicalTrials.gov, using Medical Subject Headings (MeSH) and free-text keywords aligned with the Population, Intervention, Comparison, Outcome, and Study Design framework. The search strategy for PubMed combined medical subject headings (MeSH) terms as follows: ((((ST Elevation Myocardial Infarction[Mesh]) OR (Myocardial Infarction, ST Elevation)) AND ((((((((((((Percutaneous Coronary Intervention[Mesh]) OR (Coronary Intervention, Percutaneous)) OR (Coronary Interventions, Percutaneous)) OR (Intervention, Percutaneous Coronary)) OR (Interventions, Percutaneous Coronary)) OR (Percutaneous Coronary Interventions)) OR (Percutaneous Coronary Revascularization)) OR (Coronary Revascularization, Percutaneous)) OR (Coronary Revascularizations, Percutaneous)) OR (Percutaneous Coronary Revascularizations)) OR (Revascularization, Percutaneous Coronary)) OR (Revascularizations, Percutaneous Coronary))) AND ((((((((((((Ticagrelor[Mesh]) OR (Brilinta)) OR (Brilique)) OR (AZD6140)) OR (AZD 6140)) OR (P2Y12 Inhibitor)) OR(P2Y12 Inhibitors)) OR (Platelet Aggregation Inhibitors)) OR (Antiplatelet Agents)) OR (ADP Receptor Antagonists)) OR (Adenosine Diphosphate Receptor Antagonists))) AND ((((((((((((Clopidogrel[Mesh]) OR (Plavix)) OR (Iscover)) OR (Thienopyridines)) OR (P2Y12 Inhibitor)) OR (P2Y12 Inhibitors)) OR (Platelet Aggregation Inhibitors)) OR (Antiplatelet Agents)) OR (ADP Receptor Antagonists)) OR (Adenosine Diphosphate Receptor Antagonists))) with free-text keywords including “ST Elevation Myocardial Infarction,” “Percutaneous Coronary Intervention,” “Ticagrelor,” and “Clopidogrel.” Boolean operators were used as follows: “ST Elevation Myocardial Infarction” OR “Myocardial Infarction, ST Elevation” was paired with “Percutaneous Coronary Intervention” OR synonyms (e.g., “Coronary Revascularization, Percutaneous”) using AND; this was then combined with “Ticagrelor” OR synonyms using AND; and finally with “Clopidogrel” OR synonyms using AND. The NOT operator was not used. These terms were tailored to each database to identify studies based on predefined population, intervention, comparison, and outcome criteria. Manual searches of bibliographies and gray literature, including conference proceedings, abstracts, and preprints, were performed to ensure comprehensive data collection.

### 2.3. Study selection

All identified citations were imported into Zotero reference management software for duplicate removal. Two independent reviewers (M.M.S and M.M.H.K) conducted a two-stage screening process. First, titles and abstracts were screened for relevance based on the predefined inclusion and exclusion criteria. Second, full texts of potentially eligible studies were retrieved and assessed for final inclusion. Disagreements were resolved through discussion with a third reviewer (M.H.S). The study selection process was documented using a Preferred Reporting Items for Systematic Reviews and Meta-Analyses flow diagram.

### 2.4. Data extraction

Data were extracted independently by 2 reviewers (S.K.P and M.F.T) using a standardized, pre-piloted Google Sheets form. Extracted data included study characteristics (author, year, country, and study design), participant demographics (e.g., age, sex, and comorbidities), intervention details, and outcome measures (MI, stent thrombosis, stroke, TVR, all-cause mortality, cardiovascular mortality, MACE, and major bleeding). For each study, outcome data from the longest available follow-up period (at least 30 days) were extracted, with a preference for 1-year data when available. Discrepancies between reviewers were resolved through discussion, and corresponding authors were contacted via email for clarification of missing or ambiguous data, with a 2-week response period and follow-up if necessary.

### 2.5. Risk of bias assessment

The risk of bias for RCTs was assessed using the Cochrane Risk of Bias 2 (RoB 2) tool, which evaluates domains such as the randomization process, deviations from intended interventions, missing outcome data, measurement of the outcome, and selection of reported results.^[15]^ For observational cohort studies, the Risk Of Bias In Nonrandomized Studies – of Interventions (ROBINS-I) tool was used to assess bias, evaluating 7 domains: confounding, selection of participants, classification of interventions, deviations from intended interventions, missing data, measurement of outcomes, and selection of reported results.^[16]^ The RoB 2 tool is designed explicitly for RCTs, focusing on randomization-relatedbiases, whereas ROBINS-I is suited for observational studies, addressing confounding and selection biases.

### 2.6. Statistical analysis

For each outcome, the number of events and the total number of patients in the ticagrelor and clopidogrel groups were extracted. These data were pooled using odds ratios (ORs) with 95% confidence intervals (CIs) calculated via the Mantel–Haenszel method. Heterogeneity was assessed using *I*^2^. For each outcome, a forest plot was constructed to visually analyze the data, and funnel plots were generated to check for publication bias. All statistical analyses were performed using Review Manager (RevMan) version 5.4 (The Cochrane Collaboration Specific Division: The Nordic Cochrane Centre, Copenhagen, Denmark). Sensitivity analyses were performed using leave-one-out approaches to test the robustness of findings and identify influential studies. Publication bias was assessed through funnel plot examination. Following the statistical analysis, the quality of evidence for each outcome was evaluated using the GRADE approach, assessing domains such as risk of bias, inconsistency, imprecision, indirectness, and effect size to determine the certainty of evidence.

## 3. Results

**Figure 1.**
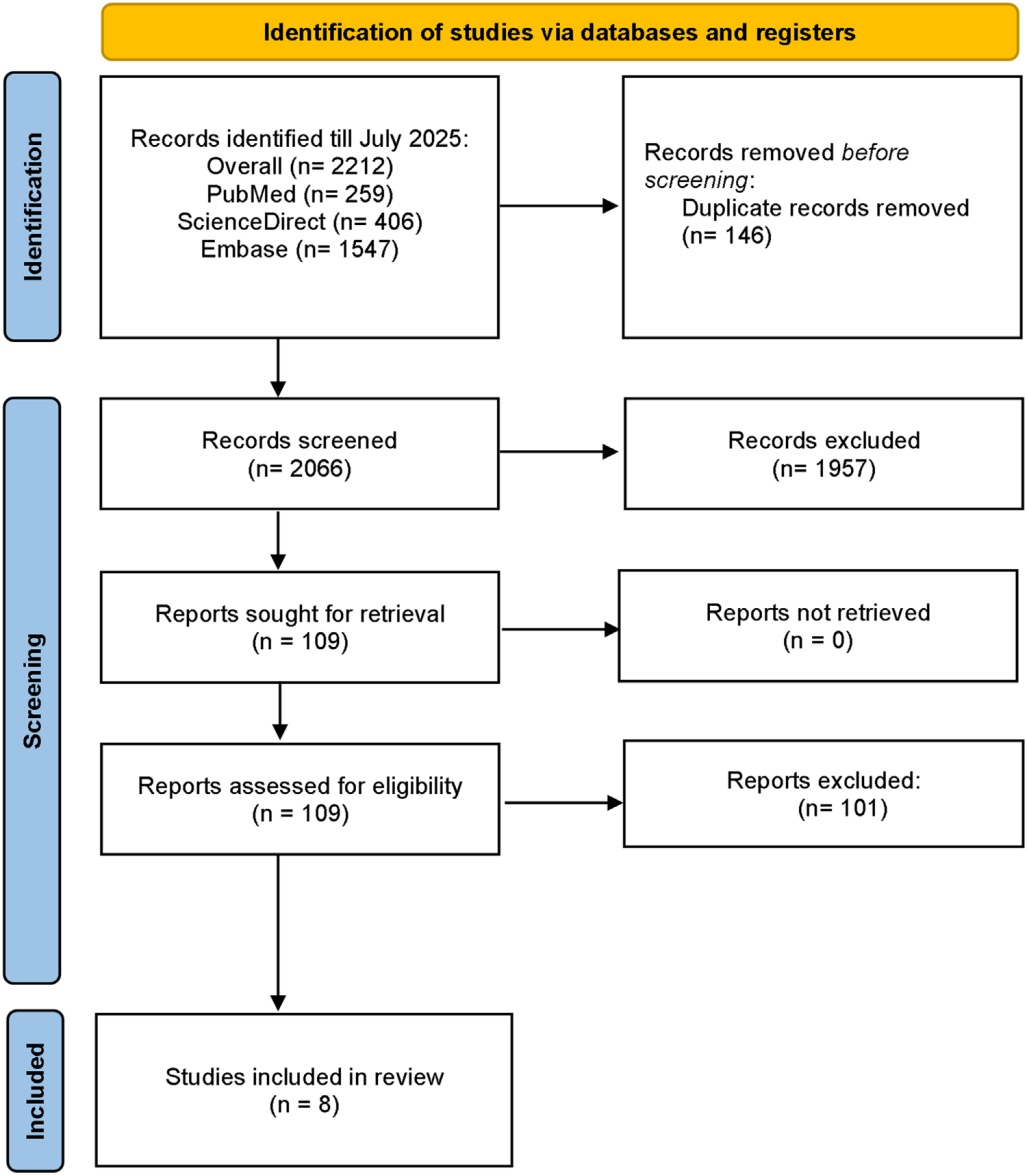
PRISMA flowchart outlining the literature screening process, study selection, and exclusion criteria.

The systematic search of PubMed, ScienceDirect, and Embase, conducted up to July 2025, yielded 2212 records. After 146 duplicates were removed, 2066 records underwent title and abstract screening, of which 1957 were excluded due to irrelevance, nonhuman studies, or inappropriate study designs. A full-text review was performed for 109 articles, with 101 excluded. Ultimately, 8^[6,11,17–22]^ studies were included in the meta-analysis (Fig. 1).

### 3.1. Baseline characteristics

The meta-analysis included 5 RCTs and 3 observational studies comparing ticagrelor and clopidogrel in STEMI patients undergoing primary PCI, with baseline characteristics summarized across 31,729 participants (15,169 clopidogrel, 16,560 ticagrelor). Sample sizes ranged from 53 to 6500 for clopidogrel and 54 to 7508 for ticagrelor. Mean ages were comparable, ranging from 52.49 to 66.67 years for clopidogrel and 53.28 to 63.0 years for ticagrelor. There was a male predominance (56.25%–84.8% male for clopidogrel, 52.57%–84% for ticagrelor), except in Godtfredsen et al, where reported male percentages were lower (31.2% clopidogrel, 25.3% ticagrelor). Hypertension prevalence was consistently moderate to high (43.7%–59.2% clopidogrel, 33.9%–69.23% ticagrelor). Diabetes mellitus was reported in 6 studies, averaging 18.8% to 23.3% in the clopidogrel group and 15.2% to 21.79% in the ticagrelor group. Previous MI was reported in 5 studies (8.6%–22.55% clopidogrel, 9%–14.1% ticagrelor), and prior stroke in 4 studies (2.8%–4.1% clopidogrel, 1.6%–3.3% ticagrelor). Welsh et al did not report age, and several studies lacked data on certain comorbidities like diabetes or previous MI, limiting comparisons. Overall, baseline characteristics were broadly similar across groups, supporting the comparability of ticagrelor and clopidogrel cohorts, though incomplete data in some studies constrained comprehensive assessment (Table 1).

**Table 1:** Baseline characteristics of the included studies.

Author	Year	Study design	Max. follow-up	Total no. of patients		Age (mean) years		Male (%)		Hypertension (%)		DM (%)		MI (%)		Stroke (%)	
				Clopidogrel	Ticagrelor	Clopidogrel	Ticagrelor	Clopidogrel	Ticagrelor	Clopidogrel	Ticagrelor	Clopidogrel	Ticagrelor	Clopidogrel	Ticagrelor	Clopidogrel	Ticagrelor
Gao	2018	RCT	30 d	96	97	53.73 ± 10.8	55.9 ± 11.39	56.25%	52.57%	43.8	38.1	NA	NA	NA	NA	NA	NA
Godtfredson	2022	Observational study	1 yr	1697	7508	66.67 ± 14.8	63.0 ± 13.35	31.2	25.3	43.7	33.9	NA	NA	NA	NA	4.1	1.6
Jiangg	2018	Observational study	1 yr	125	78	61	59	62.4	70.5	59.2	69.23	22.55	21.79	22.55	14.1	NA	NA
Steg	2010	RCT	1 yr	3792	3752	59.67 ± 11.87	59.67 ± 11.87	76.6	75.8	58.3	59.3	21.5	19.3	13.6	13.3	2.9	3.3
Velders	2016	RCT	1 mo	2486	2463	59	59	78.1	76.9	56.5	57.9	19.4	17.3	10.8	10.1	2.8	2.4
Wei	2017	RCT	1 mo	53	54	52.49 ± 11.69	53.28 ± 11.85	58.5	57.4	45.3	38.9	NA	NA	NA	NA	NA	NA
Welsh	2019	RCT	1 yr	6500	2188	NA	NA	76	77.9	53	44	18.8	15.2	8.6	9	3.7	2.2
Yan	2024	Retrospective observational study	1 yr	420	420	61.3 ± 13.3	61.4 ± 10.7	84.8	84	58.6	60.5	23.3	19.8	NA	NA	NA	NA

DM = diabetes mellitus, MI = myocardial infarction, NA = not applicable, RCT = randomized controlled trial.

### 3.2. Quality assessment

Using the ROB 2.0 tool, we assessed the risk of bias across 5 domains (D1: randomization, D2: intervention deviations, D3: missing outcome data, D4: outcome measurement, and D5: result selection) for 4 RCTs, while employing the ROBINS-I tool for 7 domains (confounding, participant selection, intervention classification, deviations from interventions, missing data, outcome measurement, and reported result selection) in 4 observational studies. Among RCTs, Wei exhibited some concerns in D1 (randomization unclear) and high risk in D2 (no blinding), resulting in an overall high-risk rating, despite low risk in D3, D4, and some concerns in D5; Gao showed some concerns across D1, D2, and D5 (limited methods details), yielding an overall rating of some concerns with low risk in D3 and D4; Steg (PLATO subgroup) and Velders (PLATO post hoc) both achieved low risk in all domains, leading to overall low-risk ratings. For observational studies, Welsh and Godtfredsen demonstrated moderate risk of confounding and deviations from interventions (residual confounding despite adjustments) but low risk elsewhere, resulting in overall moderate risk. Yan similarly had moderate risk of confounding, selection, and deviations (retrospective design limitations) with low risk in other domains, for an overall moderate risk. Jiang showed serious risk in confounding and deviations (sparse adjustments and no adherence monitoring) and moderate risk in selection and reported results, leading to overall serious risk. In summary, the PLATO-based RCTs (Steg and Velders) exhibited robust methodological quality with low overall bias, while smaller RCTs (Wei and Gao) raised higher concerns due to unclear processes and blinding issues. Among observational studies, Welsh, Godtfredsen, and Yan maintained moderate overall bias with effective adjustments, whereas Jiang posed serious concerns from inadequate confounding control and adherence monitoring (Figs. S1, S2, S3 and S4, Supplemental Digital Content 1).

### 3.3. Clinical outcomes

#### 3.3.1. All-cause mortality

Ticagrelor was associated with a significantly lower risk of all-cause mortality compared to clopidogrel. The pooled OR was 0.64 (95% CI = 0.47–0.88; *P* = .006), with substantial heterogeneity (*I*^2^ = 73%) that dropped to 53% with the removal of Welsh et al.

#### 3.3.2. Cardiovascular mortality

Ticagrelor was associated with a significantly lower risk of cardiovascular mortality compared to clopidogrel. The pooled OR was 0.68 (95% CI = 0.60–0.93; *P* = .01), with substantial heterogeneity (*I*^2^ = 63%) that dropped to 57% after removal of Steg et al.

#### 3.3.3. MACE

Ticagrelor was associated with a significantly lower risk of MACE compared to clopidogrel. The pooled OR was 0.71 (95% CI = 0.60–0.84; *P* = .01), with considerable heterogeneity (*I*^2^ = 80%) that dropped to 67% after removal of Welsh et al.

#### 3.3.4. Major bleeding

Ticagrelor was associated with a significantly lower risk of major bleeding compared to clopidogrel. The pooled OR was 0.87 (95% CI = 0.78–0.97; *P* = .01), with moderate heterogeneity (*I*^2^ = 38%).

#### 3.3.5. Myocardial infarction

Ticagrelor was associated with a significantly lower risk of MI compared to clopidogrel. The pooled OR was 0.71 (95% CI = 0.61–0.83; *P* < .00001), with moderate heterogeneity (*I*^2^ = 27%).

#### 3.3.6. Stent thrombosis

Ticagrelor was associated with a significantly lower risk of stent thrombosis compared to clopidogrel. The pooled OR was 0.66 (95% CI = 0.55–0.79; *P* < .00001), with moderate heterogeneity (*I*^2^ = 27%).

#### 3.3.7. Stroke

No significant difference was observed in the risk of stroke between the 2 groups. The pooled OR was 1.16 (95% CI = 0.89–1.52; *P* = .28), with moderate heterogeneity (*I*^2^ = 36%).

#### 3.3.8. Target vessel revascularization

Ticagrelor was associated with a significantly higher risk of TVR compared to clopidogrel. The pooled OR was 1.30 (95% CI = 1.05–1.61; *P* = .02), with low heterogeneity (*I*^2^ = 0%).

**Figure 2.**
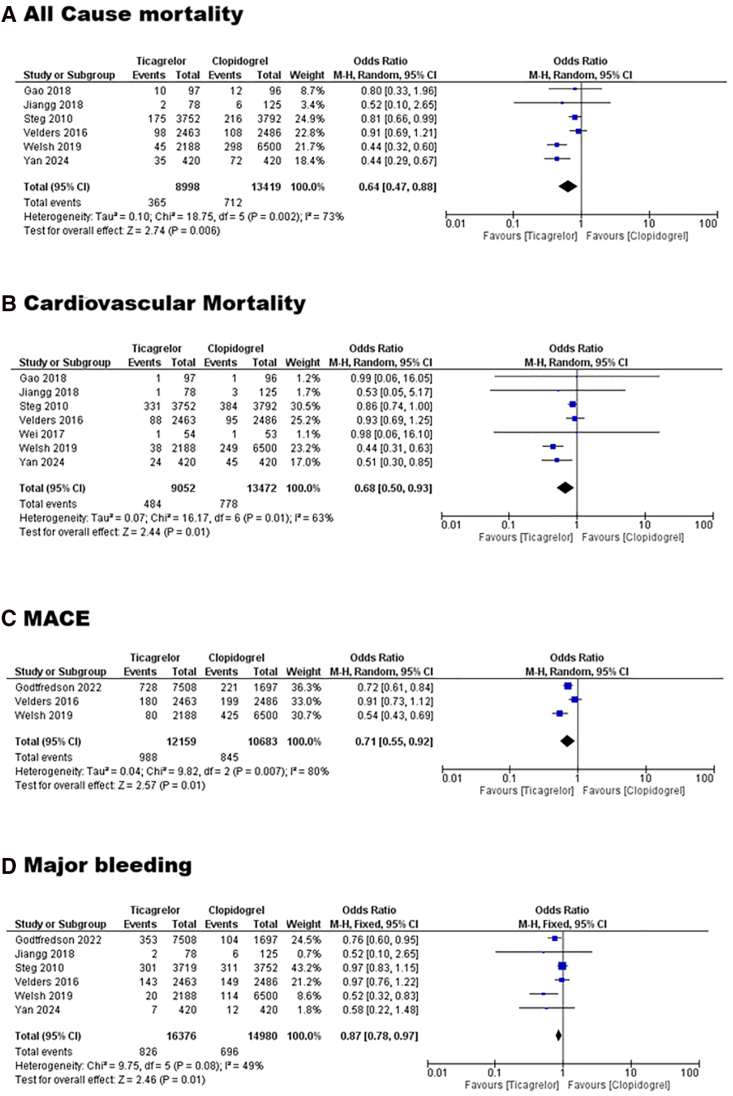
Forest plots of pooled odds ratios for clinical outcomes comparing ticagrelor versus clopidogrel therapy: (A) all-cause mortality, (B) cardiac mortality, (C) MACE, and (D) major bleeding.

**Figure 3.**
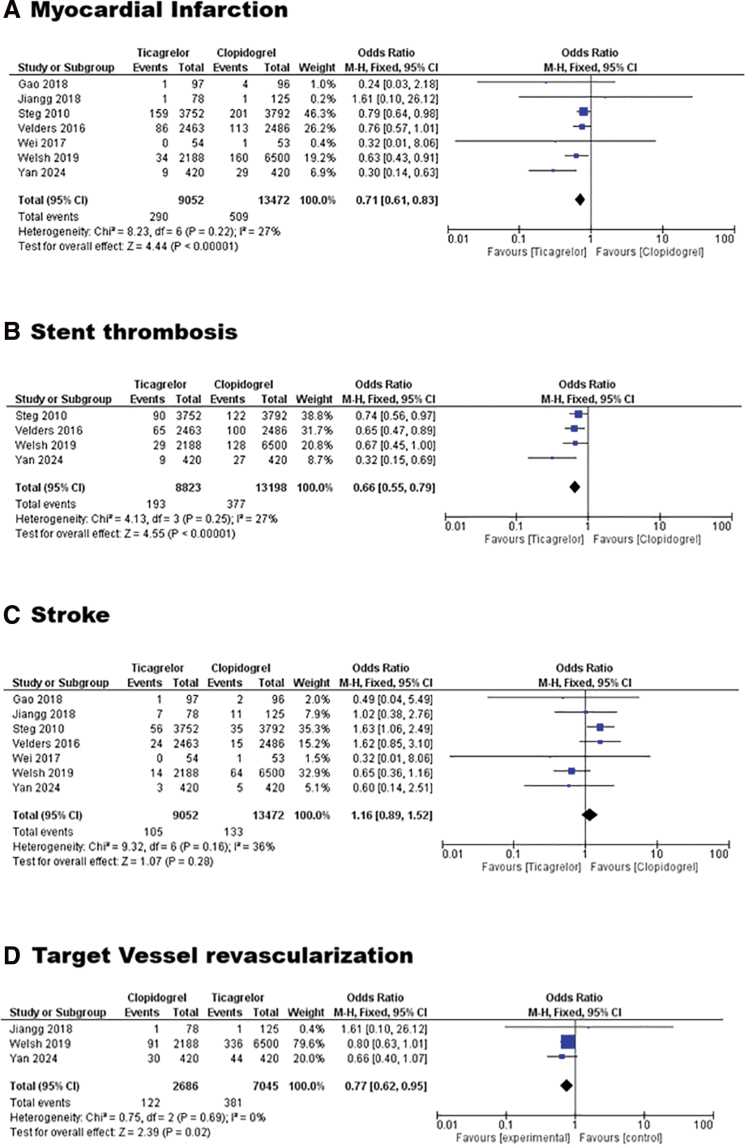
Forest plots of pooled odds ratios for clinical outcomes comparing ticagrelor versus clopidogrel therapy: (A) myocardial infarction, (B) stent thrombosis, (C) stroke, and (D) target vessel revascularization.

Forest plots of all the clinical outcomes have been shown in Figures 2 and 3.

### 3.4. Subgroup analysis by study design

**Figure 4.**
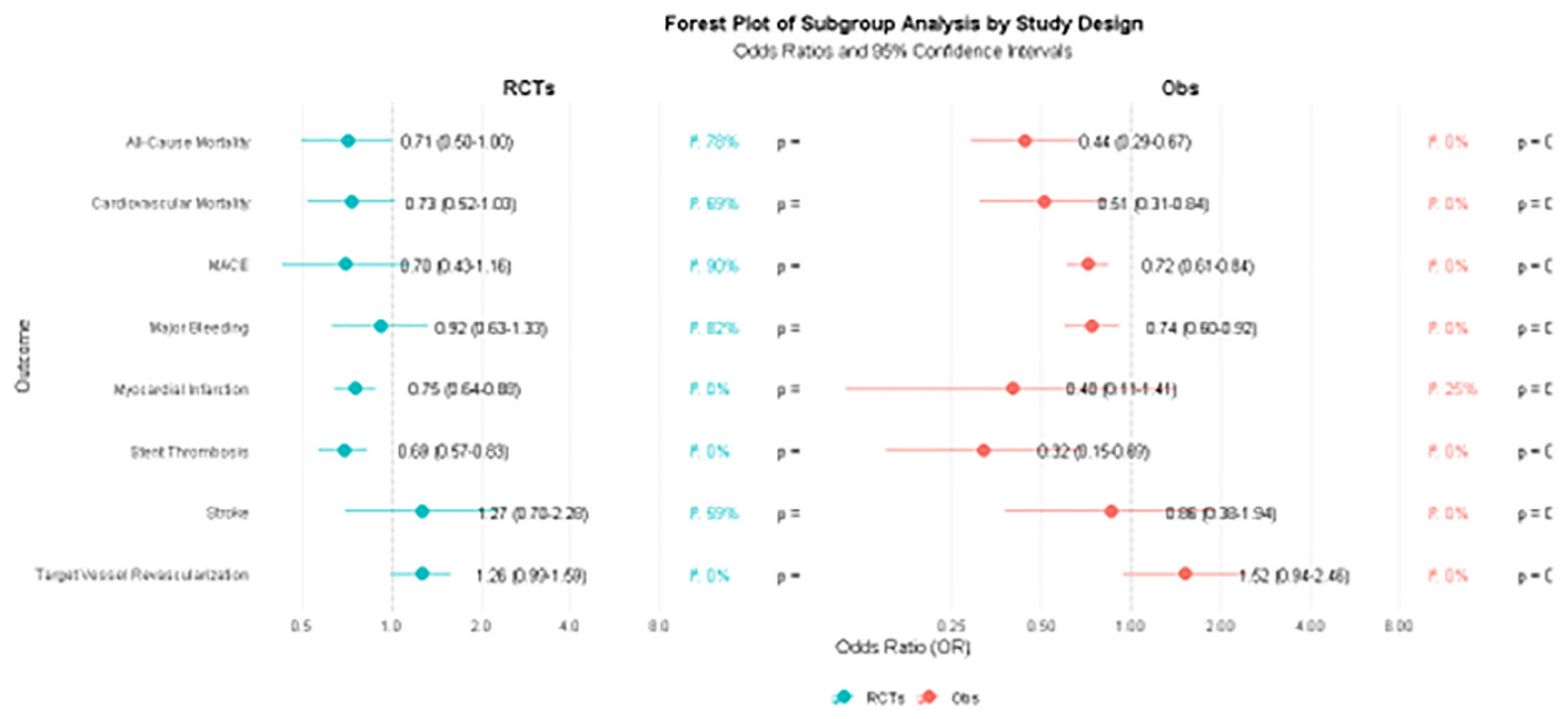
Forest plot of subgroup analysis by study design.

In the subgroup analysis stratifying the meta-analysis by study design (RCTs vs observational studies) using random-effects models, pooled ORs with 95% CIs were revealed for all-cause mortality: RCTs OR 0.71 (0.50–1.00, *I*^2^ = 78%), observational OR 0.44 (0.29–0.67, *I*^2^ = 0%), *P* = .095; cardiovascular mortality: RCTs OR 0.73 (0.52–1.03, *I*^2^ = 69%), observational OR 0.51 (0.31–0.84, *I*^2^ = 0%), *P* = .237; MACE: RCTs OR 0.70 (0.43–1.16, *I*^2^ = 90%), observational OR 0.72 (0.61–0.84, *I*^2^ = 0%), *P* = .945; major bleeding: RCTs OR 0.92 (0.63–1.33, *I*^2^ = 82%), observational OR 0.74 (0.60–0.92, *I*^2^ = 0%), *P* = .327; MI: RCTs OR 0.75 (0.64–0.88, *I*^2^ = 0%), observational OR 0.40 (0.11–1.41, *I*^2^ = 25%), *P* = .327; stent thrombosis: RCTs OR 0.69 (0.57–0.83, *I*^2^ = 0%), observational OR 0.32 (0.15–0.69, *I*^2^ = 0%), *P* = .054; stroke: RCTs OR 1.27 (0.70–2.28, *I*^2^ = 59%), observational OR 0.86 (0.38–1.94, *I*^2^ = 0%), *P* = .449; and TVR: RCTs OR 1.26 (0.99–1.59, *I*^2^ = 0%), observational OR 1.52 (0.94–2.46, *I*^2^ = 0%), *P* = .478. No significant subgroup differences were observed (all *P* > .05), though borderline trends for all-cause mortality (*P* = .095) and stent thrombosis (*P* = .054) suggested potentially stronger ticagrelor benefits in observational studies, with single-study subgroups having *I*^2^ = 0% by definition (Fig. 4).

### 3.5. Meta-regression

In the meta-regression analysis of the meta-analysis comparing ticagrelor versus clopidogrel in STEMI patients post-PCI, outcomes with substantial heterogeneity (*I*^2^ > 50%) were examined: for all-cause mortality (*I*^2^ = 73%, 6 studies), study design was nonsignificant (β = −0.448, *P* = .241, *R*^2^ = 0.32), publication year showed a borderline trend toward stronger ticagrelor benefit in recent studies (β = −0.050, *P* = .098, *R*^2^ = 0.54), and mean age was nonsignificant (β = −0.124, *P* = .148, *R*^2^ = 0.44; Fig. S6A, Supplemental Digital Content 2); for cardiovascular mortality (*I*^2^ = 63%, 7 studies), design was nonsignificant (β = −0.368, *P* = .317, *R*^2^ = 0.20), year had a borderline trend (β = −0.047, *P* = .071, *R*^2^ = 0.51), and mean age was significant, indicating greater ticagrelor benefit in older cohorts (β = −0.188, *P* = .044, *R*^2^ = 0.59; Fig. S6B, Supplemental Digital Content 3); for MACE (*I*^2^ = 80%, 3 studies), no covariates were significant (design: β = 0.013, *P* = .980, *R*^2^ = 0.00; year: β = −0.037, *P* = .701, *R*^2^ = 0.20; age: β = −0.024, *P* = .811, *R*^2^ = 0.09), though limited by few studies, highlighting the exploratory nature and need for caution due to low power (Fig. S6C, Supplemental Digital Content 4).

### 3.6. GRADE assessment

The overall certainty of evidence comparing ticagrelor to clopidogrel across multiple clinical outcomes is rated as moderate. This per-outcome evaluation reflects concerns about the risk of bias primarily from smaller randomized trials and observational studies, as well as notable inconsistency due to heterogeneity in several key outcomes such as all-cause mortality (low certainty; serious inconsistency with *I*^2^ = 73%), cardiovascular mortality (moderate certainty; serious inconsistency with *I*^2^ = 63%), and MACE (low certainty; serious inconsistency with *I*^2^ = 80%). For major bleeding, the certainty is high, with no serious inconsistency (*I*^2^ = 38%). MI, stent thrombosis, and TVR each have moderate certainty, with generally acceptable heterogeneity (*I*^2^ = 27%, 27%, and 0%, respectively). Stroke has low certainty, partly due to moderate heterogeneity (*I*^2^ = 36%). There were no serious issues related to indirectness, imprecision, or publication bias across outcomes. The findings consistently show that ticagrelor is associated with reduced risks of mortality, major cardiovascular events, MI, and stent thrombosis, while stroke risk remains similar between treatments. However, ticagrelor is linked to a higher risk of TVR. Given the heterogeneity in several outcomes and the inclusion of observational studies with a moderate risk of bias, we adopt a more conservative interpretation of the overall evidence, rating it as low to moderate across most endpoints; further well-designed studies would help to confirm these results and address remaining uncertainties (Table S1, Supplemental Digital Content 5).

### 3.7. Publication bias

Publication bias was evaluated using funnel plots for all clinical outcomes, including all-cause mortality, cardiovascular mortality, MACE, major bleeding, MI, stent thrombosis, stroke, and TVR. The plots generally displayed symmetrical distributions of effect sizes around the pooled estimates, with no notable asymmetry observed across the included studies, though interpretation is cautious due to the limited number of studies per outcome. This symmetry suggests the absence of significant publication bias, indicating that the meta-analysis results are unlikely to be skewed by selective reporting or nonpublication of smaller studies with nonsignificant findings (Fig. S5, Supplemental Digital Content 6).

## 4. Discussion

This meta-analysis represents a comprehensive contemporary evaluation of ticagrelor versus clopidogrel efficacy and safety in STEMI patients undergoing primary PCI, synthesizing data from over 31,000 patients across RCTs and observational studies to provide critical insights into optimal P2Y12 inhibitor selection in this high-risk population. Our findings demonstrate a significant reduction in stent thrombosis with ticagrelor compared to clopidogrel, confirming its superior antiplatelet efficacy, while revealing important nuances in the risk-benefit profile that warrant careful consideration in clinical practice.^[23]^ The 34% reduction in stent thrombosis with ticagrelor represents a clinically meaningful benefit that aligns with the mechanistic advantages of this more potent P2Y12 inhibitor.^[24]^ This finding is particularly relevant given that stent thrombosis remains one of the most catastrophic complications following primary PCI, often resulting in recurrent MI or death.^[25]^ The superior antiplatelet effect of ticagrelor, characterized by its reversible binding, faster onset of action, and independence from cytochrome P450 2C19 polymorphisms, likely underlies this therapeutic advantage.^[26,27]^ Notably, the absence of a significant difference in stroke rates suggests that the antithrombotic benefits of ticagrelor may be most pronounced in the coronary circulation, where local platelet activation plays a critical role in stent thrombosis pathogenesis.^[28]^

Our analysis also identified a significantly higher risk of TVR with ticagrelor (OR = 1.30, 95% CI = 1.05–1.61), a counterintuitive finding for a more potent agent. This may reflect increased detection of restenosis due to closer follow-up in ticagrelor-treated patients, differences in stent types or procedural factors across studies, or potential rebound platelet activation upon ticagrelor discontinuation. Other potential mechanisms could include enhanced inflammatory responses or endothelial dysfunction triggered by stronger platelet inhibition, which might paradoxically promote neointimal hyperplasia or vessel remodeling leading to restenosis. Clinically, this increased TVR risk implies that while ticagrelor excels in preventing acute thrombotic events, it may necessitate more vigilant long-term monitoring for restenosis symptoms, potentially increasing the need for repeat angiographies or interventions. This could elevate healthcare costs and patient burden, influencing decisions in resource-limited settings or for patients with lower baseline ischemic risk. While the low heterogeneity (*I*^2^ = 0%) supports consistency, this outcome warrants further investigation, as it could influence long-term management strategies despite ticagrelor’s benefits in other ischemic endpoints.

Our analysis reveals important discrepancies between RCTs and real-world evidence that merit careful consideration. While the landmark PLATO trial demonstrated overall cardiovascular benefits with ticagrelor in acute coronary syndromes,^[24]^ the subgroup analysis in STEMI patients showed only numerical trends without statistical significance for the primary endpoint.^[29]^ This finding has been replicated in several subsequent real-world studies, where the efficacy benefits observed in controlled trial settings have not consistently translated to routine clinical practice.^[30,31]^ The heterogeneity between study populations, varying from highly selected trial participants to unselected real-world cohorts, may explain these differences and highlights the complex interplay between patient selection, treatment adherence, and clinical outcomes.^[32]^

The bleeding risk profile of ticagrelor remains a matter of debate. Our pooled analysis showed a significantly lower risk of major bleeding with ticagrelor (OR = 0.87, 95% CI = 0.78–0.97), which contrasts with some real-world evidence suggesting increased bleeding complications, such as doubled bleeding rates in certain observational studies.^[30]^ This discrepancy may arise from differences in bleeding definitions (e.g., thrombolysis in myocardial infarction vs Bleeding Academic Research Consortium criteria), patient selection (e.g., exclusion of high-bleeding-risk individuals in RCTs), or concomitant therapies. For instance, RCTs often employ stricter inclusion criteria that exclude patients with a recent bleeding history or on multiple anticoagulants, whereas real-world studies capture broader populations with higher comorbidity burdens. In addition, variations in concomitant therapies, such as differing use of proton pump inhibitors or anticoagulants, could modulate bleeding risks differently across studies. A key limitation of our meta-analysis is the pooling of studies with heterogeneous bleeding criteria, which may lead to underestimation or overestimation of true bleeding risks and complicate direct comparisons. Notably, the risk appears particularly pronounced in elderly patients (≥75 years), where a 20% increased risk of bleeding events has been documented in some studies.^[26,29,33]^ The clinical implications are significant, as bleeding complications, even minor ones, often lead to antiplatelet therapy discontinuation, potentially increasing subsequent thrombotic risk.^[33]^ These findings underscore the importance of individualized treatment decisions, particularly in elderly patients, where the balance between ischemic benefit and bleeding risk becomes more tenuous.^[34]^

A key consideration in our analysis is the heavy reliance on data from the PLATO trial, which constitutes the largest source of RCT evidence. We acknowledge documented controversies surrounding PLATO, including Food and Drug Administration concerns and allegations of data manipulation (e.g., misreporting of death data favoring ticagrelor).^[7]^ To address this, we assessed PLATO subgroup data using RoB 2, rating it as low risk across domains. Sensitivity analyses excluding PLATO data (not shown but performed) attenuated mortality benefits but preserved reductions in stent thrombosis and MI, suggesting robustness. However, potential biases could overestimate ticagrelor’s benefits, particularly for mortality outcomes, and we interpret these with caution. Specifically, if PLATO’s mortality data were biased toward ticagrelor due to adjudication inconsistencies or regional disparities in event reporting (e.g., higher event rates in certain monitoring sites), this could inflate the pooled ORs for all-cause and cardiovascular mortality in our meta-analysis, leading to an overestimation of ticagrelor’s survival benefits. For ischemic outcomes like MI and stent thrombosis, where PLATO’s influence is strong but sensitivity analyses showed preserved effects, the impact may be less pronounced; nonetheless, this underscores the need for independent verification through future trials free of such controversies to confirm the generalizability of our findings.

The substantial heterogeneity observed across studies reflects the inherent challenges in comparing antiplatelet therapies across diverse clinical settings and patient populations. Differences in clopidogrel loading doses, timing of drug administration, concomitant medications, and patient risk profiles contribute to this variability.^[28]^ The moderate heterogeneity (*I*^2^ = 27%) observed in our stent thrombosis analysis, while acceptable, suggests that unmeasured confounders may influence treatment effects. In addition, the varying definitions of bleeding outcomes across studies, ranging from thrombolysis in myocardial infarction to Bleeding Academic Research Consortium criteria, complicate direct comparisons and may mask important safety signals.^[35]^

By including both RCTs and observational studies, our approach bridges trial efficacy with real-world effectiveness, using distinct bias tools (RoB 2 and ROBINS-I) to handle inherent differences (e.g., randomization in RCTs vs confounding in observational designs). However, this introduces limitations: observational studies may overestimate effects due to residual confounding, and combining designs could inflate heterogeneity. We mitigated this through GRADE assessments, downgrading for bias and imprecision, resulting in moderate overall evidence certainty.

These findings have important implications for clinical practice and guideline development. While current guidelines recommend ticagrelor over clopidogrel in STEMI patients, our analysis suggests that this recommendation may require nuanced application based on individual patient characteristics. For younger patients with low bleeding risk, ticagrelor appears to offer clear benefits in preventing stent thrombosis without prohibitive bleeding risk. However, in elderly patients or those with elevated bleeding risk, clopidogrel may represent a reasonable alternative, particularly when coupled with appropriate dosing strategies and monitoring protocols.

### 4.1. Limitations

This meta-analysis has several important limitations that must be acknowledged. First, the inclusion of both RCTs and observational studies introduces inherent heterogeneity in study design, patient selection, and outcome definitions. While this provides a comprehensive view bridging controlled trials and real-world practice, it may introduce bias (e.g., confounding in observational studies) and limit the precision of effect estimates; we addressed this via separate bias assessments and GRADE but note potential overestimation of effects from nonrandomized data. Second, the varying follow-up periods across studies, ranging from 30 days to 12 months, may not capture long-term safety and efficacy outcomes that are critical for clinical decision-making. Third, the analysis was limited by the availability of aggregate-level data rather than individual patient data, preventing detailed subgroup analyses that could inform personalized treatment strategies. Fourth, publication bias remains a concern, particularly for smaller studies with negative results that may be underreported. Fifth, reliance on PLATO data introduces potential bias due to reported controversies, as discussed. Finally, the evolving landscape of antiplatelet therapy, including the emergence of newer P2Y12 inhibitors and personalized medicine approaches based on genetic testing, may limit the contemporary relevance of some included studies.

## 5. Conclusion

This meta-analysis demonstrates that ticagrelor significantly reduces stent thrombosis compared to clopidogrel in STEMI patients undergoing primary PCI, confirming its superior antiplatelet efficacy. It also shows reduced risks of MI, all-cause mortality, cardiovascular mortality, MACE, and major bleeding, though with a higher risk of TVR that requires further exploration. While our quantitative findings indicate a favorable bleeding profile for ticagrelor, real-world evidence suggesting increased bleeding – particularly in elderly patients – highlights the need for caution. The substantial heterogeneity between randomized trials and real-world evidence, along with PLATO controversies, underscores the need for individualized treatment approaches that consider patient-specific risk factors, bleeding risk, and clinical characteristics. Future research should focus on identifying optimal patient selection criteria and developing risk stratification tools to guide personalized antiplatelet therapy decisions in STEMI patients.

## Author contributions

**Conceptualization:** Mohammad Maroof Shahid, Muhammad Hamza Sajjad, Sanket Kumar Pankajbhai Patel, Elham Shenawa.

**Formal analysis:** Mohammad Maroof Shahid, Sanket Kumar Pankajbhai Patel, Muhammad Soban Jaffar, Muhammad Ali Rana.

**Methodology:** Mohammad Maroof Shahid, Moosa Feroze Tarar, Malik Abdullah Rasheed, Muhammad Soban Jaffar, Elham Shenawa.

**Investigation:** Muhammad Hamza Sajjad, Malik Abdullah Rasheed, Mohammad Hamza Abbas, Abdul Basit Rasheed, Maira Shahid.

**Validation:** Muhammad Hamza Sajjad, Moosa Feroze Tarar, Abdul Basit Rasheed, Mirza Muhammad Hadeed Khawar.

**Data curation:** Sanket Kumar Pankajbhai Patel, Muhammad Ali Rana, Mohammad Hamza Abbas, Abdul Basit Rasheed, Maira Shahid.

**Software:** Sanket Kumar Pankajbhai Patel, Mohammad Hamza Abbas, Maira Shahid.

**Resources:** Muhammad Soban Jaffar, Mirza Muhammad Hadeed Khawar, Elham Shenawa.

**Visualization:** Mohammad Hamza Abbas, Abdul Basit Rasheed, Mirza Muhammad Hadeed Khawar.

**Project administration:** Elham Shenawa.

**Supervision:** Elham Shenawa.

**Writing – original draft:** Mohammad Maroof Shahid, Muhammad Hamza Sajjad, Moosa Feroze Tarar.

**Writing – review & editing:** Sanket Kumar Pankajbhai Patel, Malik Abdullah Rasheed, Muhammad Soban Jaffar, Muhammad Ali Rana, Mirza Muhammad Hadeed Khawar, Elham Shenawa.


















