# Supplementary figures and images for: Ticagrelor versus clopidogrel in STEMI post-PCI: A mixed-design meta-analysis of efficacy and safety
Source: Medicine (Baltimore). 2026 Jul 24;105(30):e49923. doi: 10.1097/MD.0000000000049923 (PMC13406323; doi:10.1097/MD.0000000000049923)

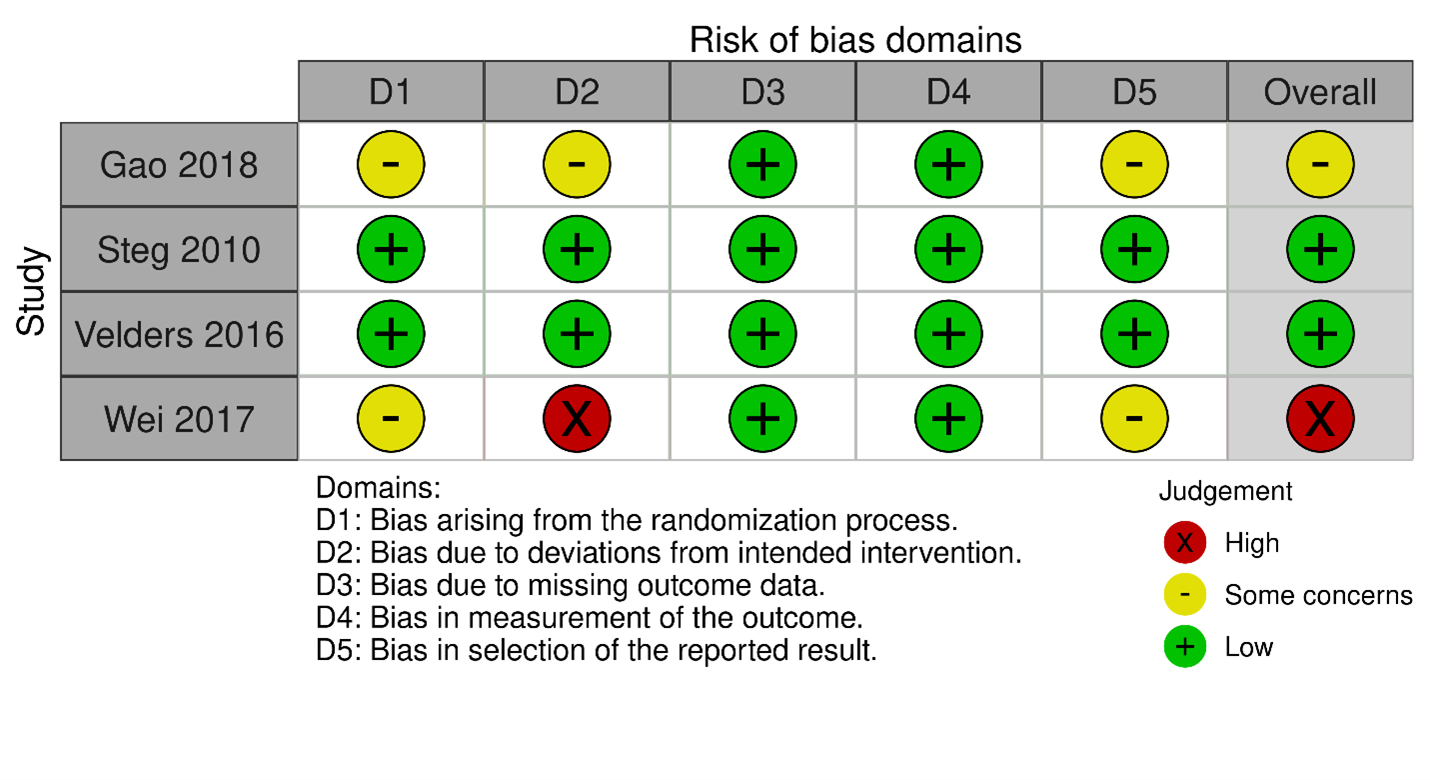

Supplement: Supplementary file 1 [file medi-105-e49923-s001.tif]

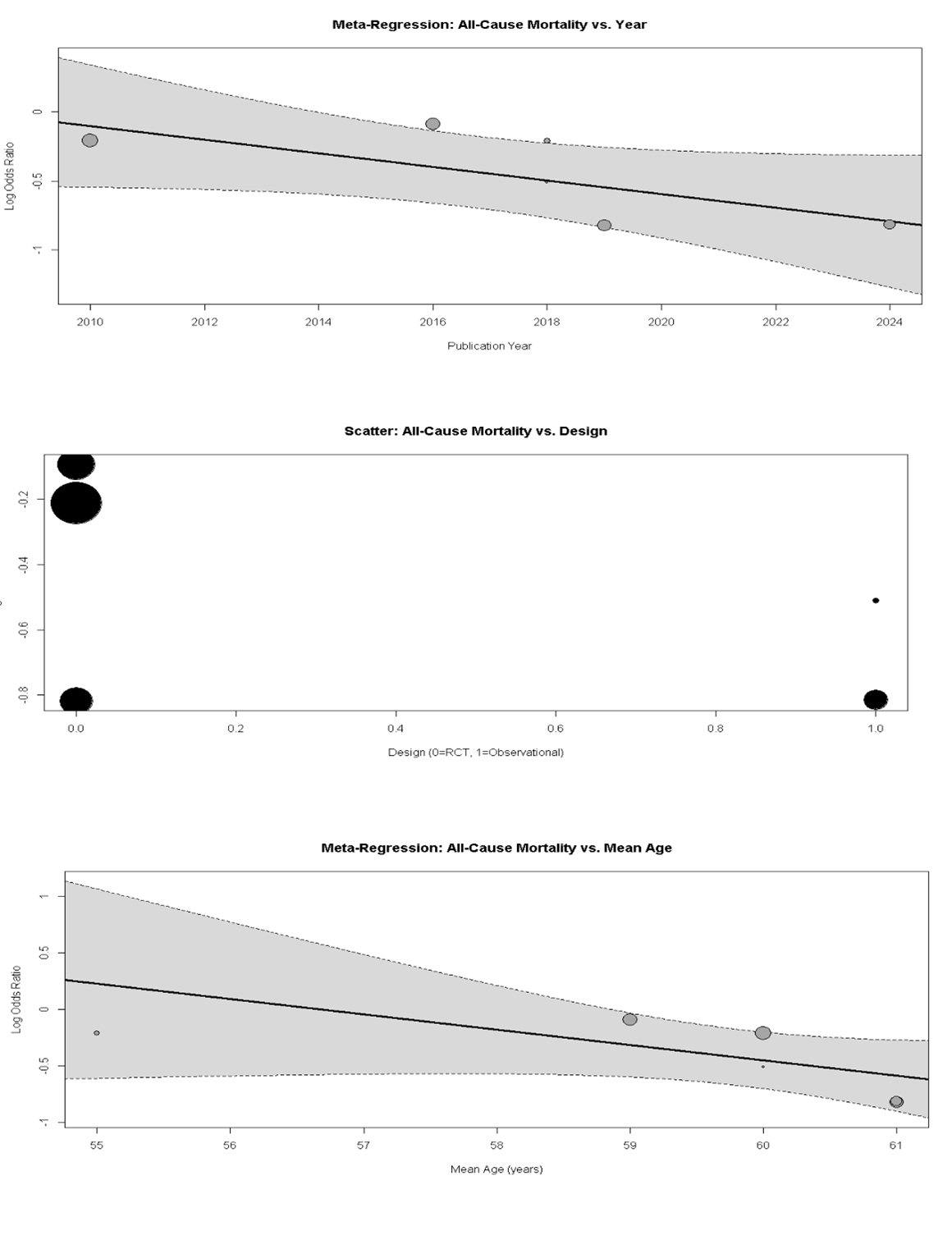

Supplement: Supplementary file 2 [file medi-105-e49923-s002.tif]

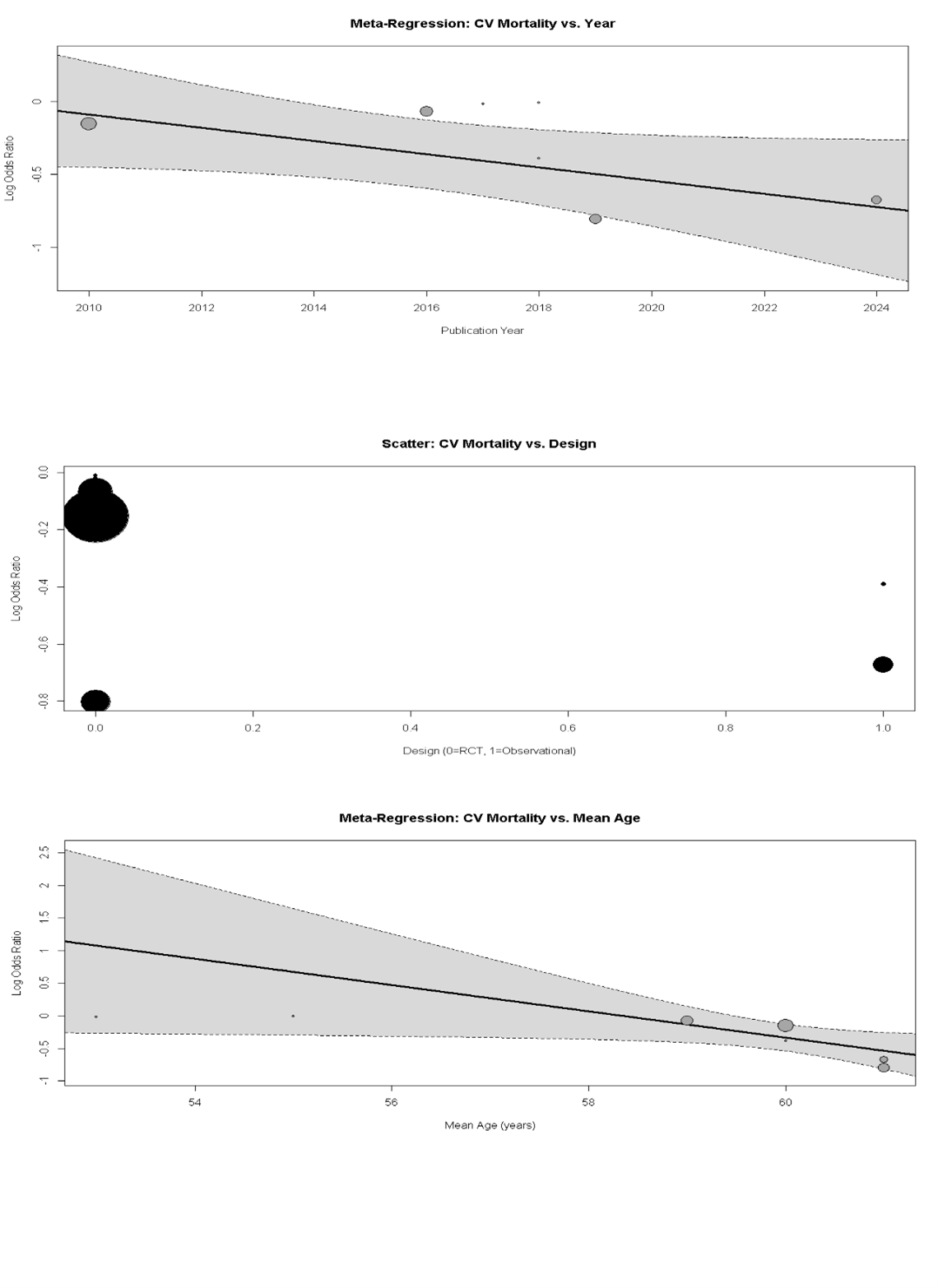

Supplement: Supplementary file 3 [file medi-105-e49923-s003.tif]

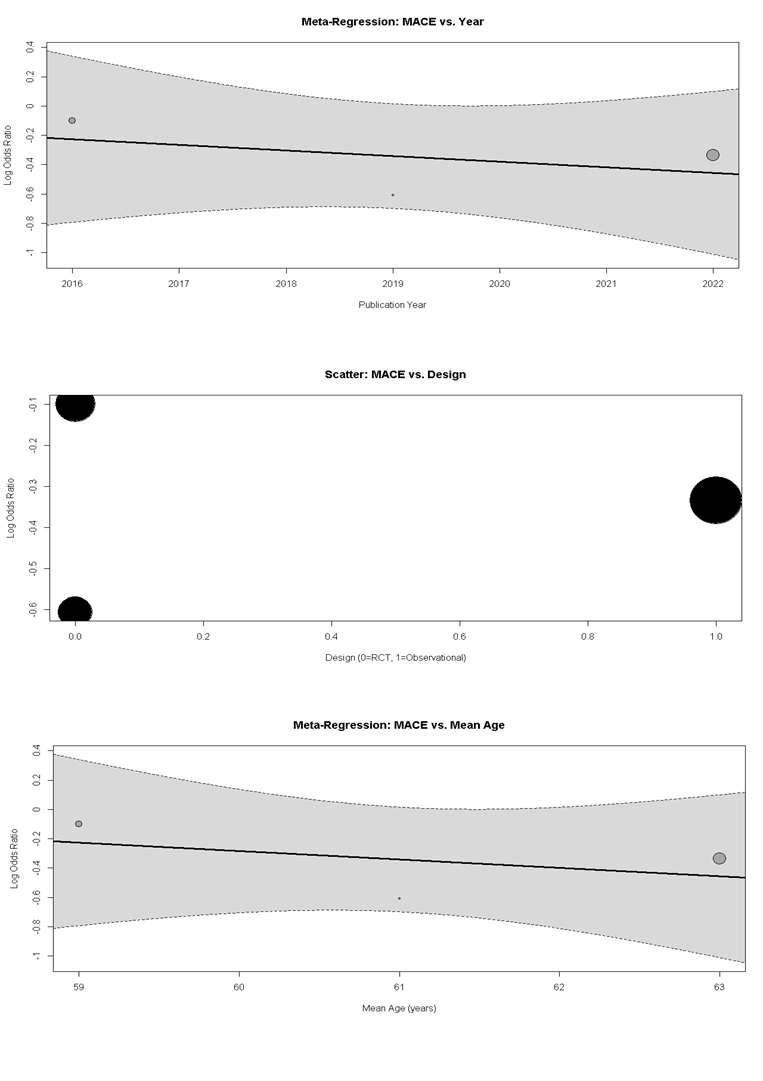

Supplement: Supplementary file 4 [file medi-105-e49923-s004.tif]

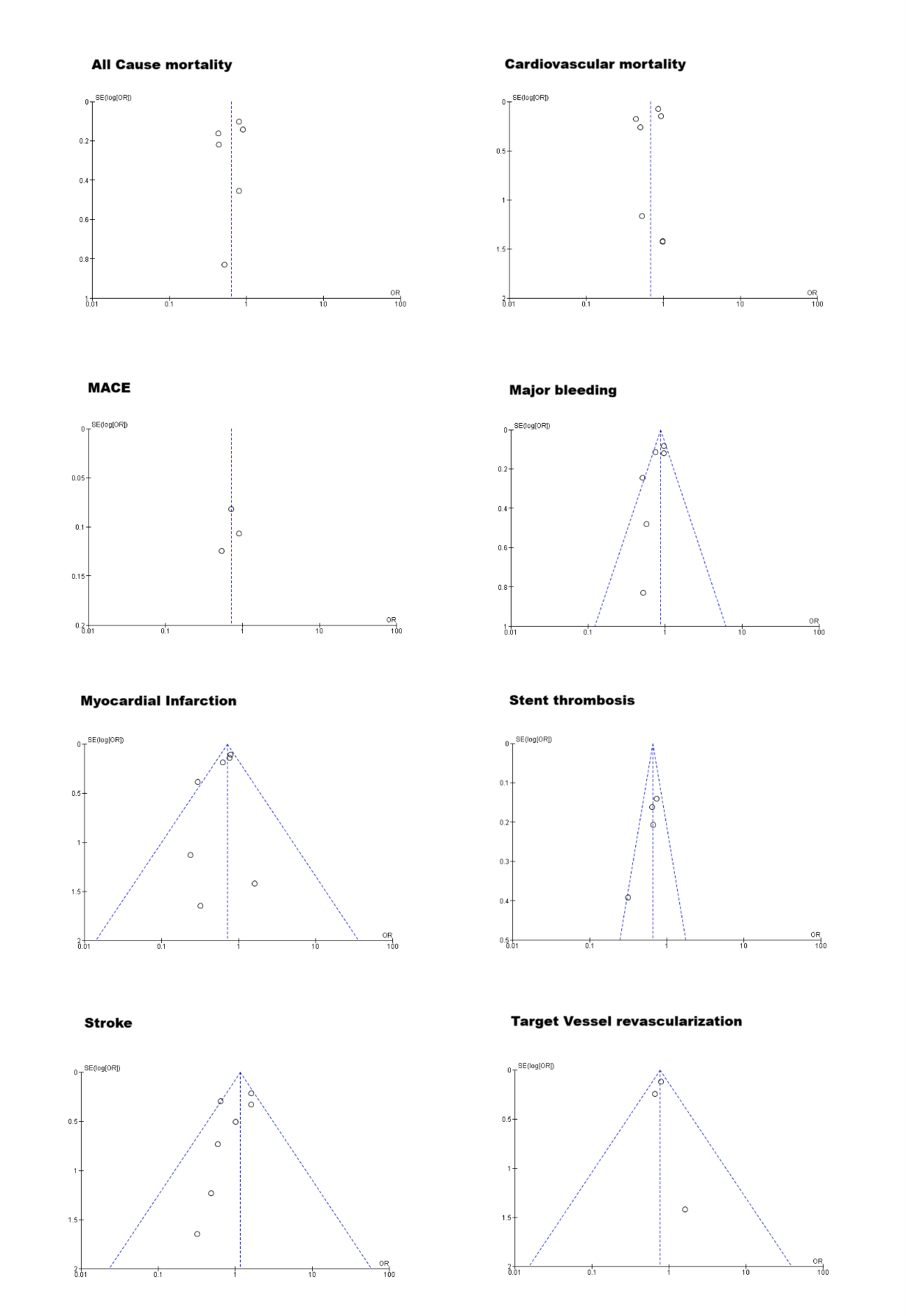

Supplement: Supplementary file 6 [file medi-105-e49923-s006.tif]

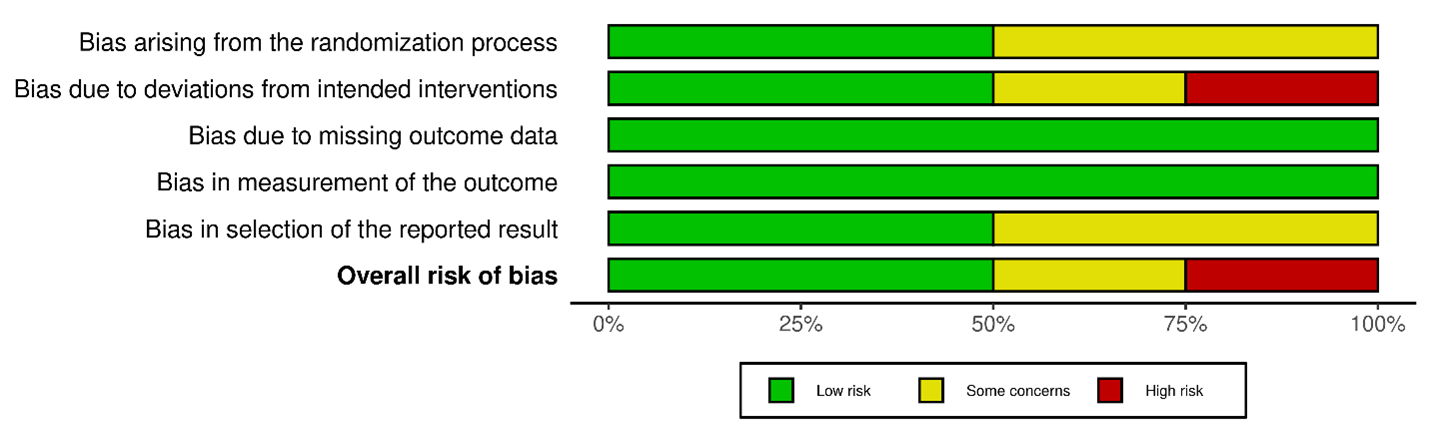

Supplement: Supplementary file 7 [file medi-105-e49923-s007.tif]

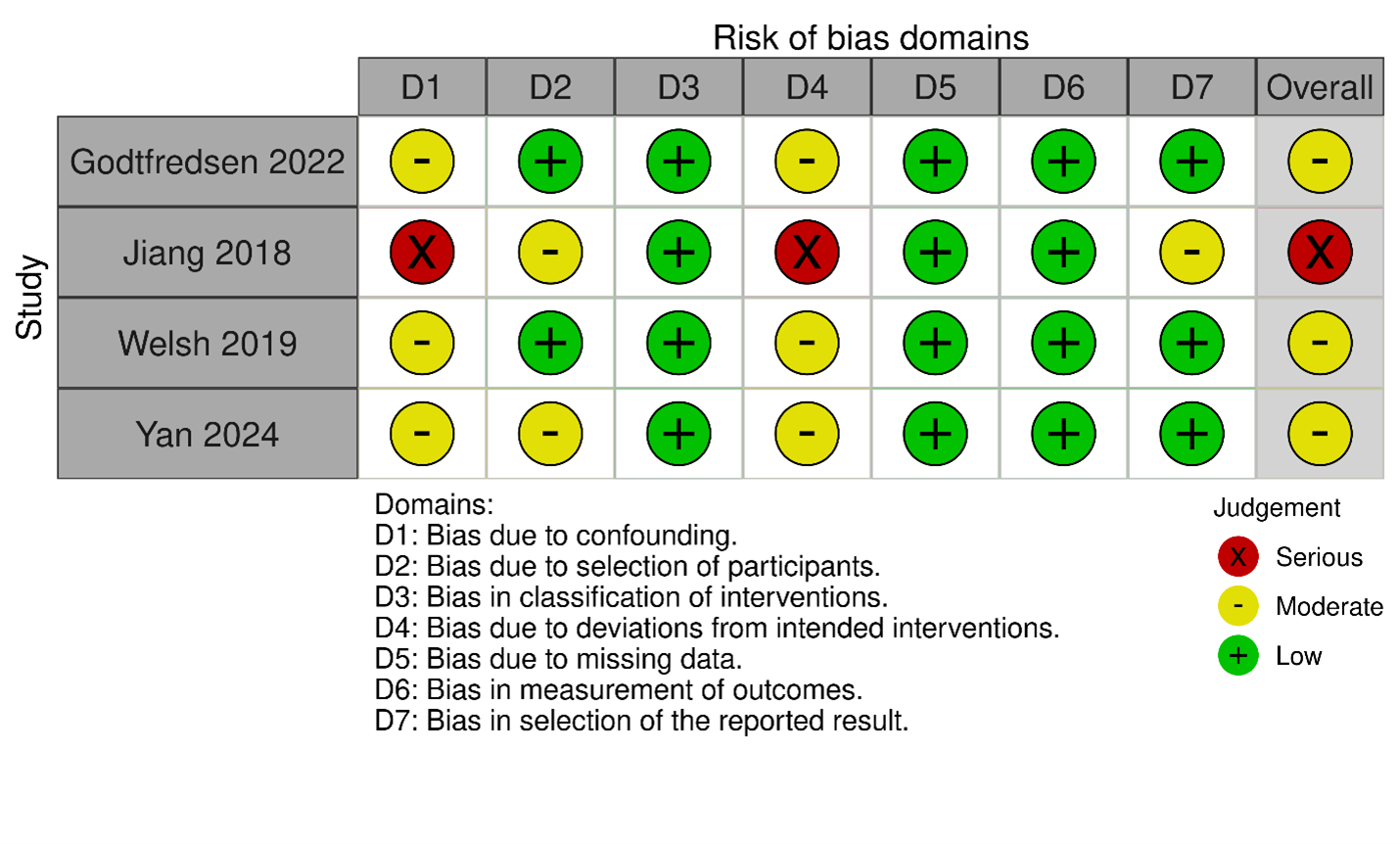

Supplement: Supplementary file 8 [file medi-105-e49923-s008.tif]

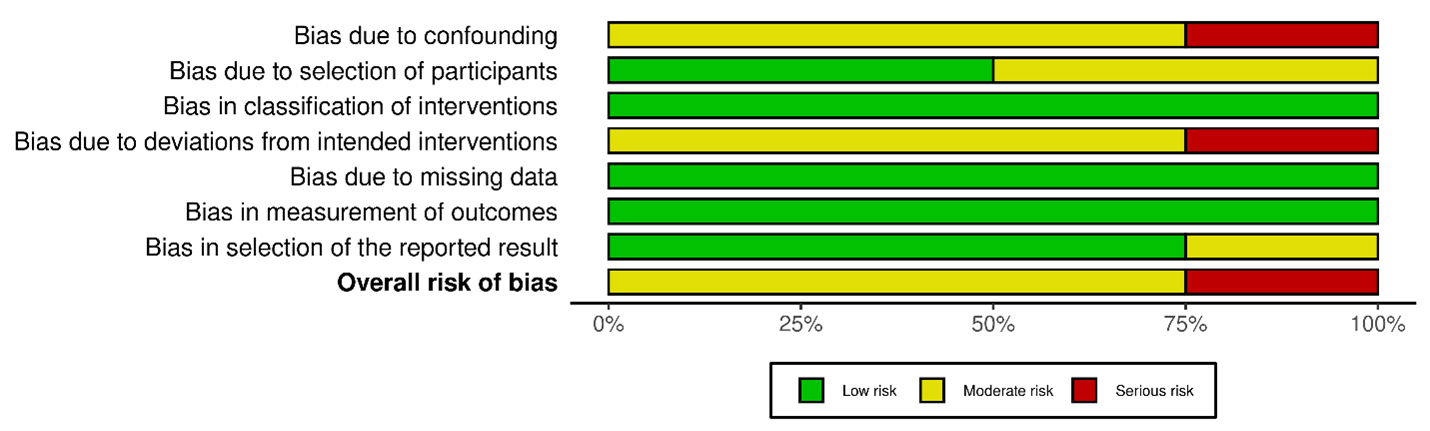

Supplement: Supplementary file 9 [file medi-105-e49923-s009.tif]
