# Supplementary material for: Ticagrelor versus clopidogrel in STEMI post-PCI: A mixed-design meta-analysis of efficacy and safety
Source: Medicine (Baltimore). 2026 Jul 24;105(30):e49923. doi: 10.1097/MD.0000000000049923 (PMC13406323; doi:10.1097/MD.0000000000049923)
Supplement: Supplementary file 5 [file medi-105-e49923-s005.docx]

**Supplementary Table 1 GRADE Assessment Table for Clinical Outcomes Comparing Ticagrelor vs. Clopidogrel**

| **Outcome** | **Risk of Bias** | **Inconsistency** | **Indirectness** | **Imprecision** | **Publication Bias** | **Overall Certainty of Evidence** |
| --- | --- | --- | --- | --- | --- | --- |
| All-Cause Mortality | Moderate (some concerns in some RCTs and observational studies) | Serious (I²=73% substantial; reduced but still moderate heterogeneity after sensitivity) | No serious concerns | No serious concerns | None detected | Low |
| Cardiovascular Mortality | Moderate (similar to all-cause mortality) | Serious (I²=63% substantial; moderate after sensitivity) | No serious concerns | No serious concerns | None detected | Moderate |
| Major Adverse Cardiovascular Events (MACE) | Moderate (some concerns in smaller RCTs and observational studies) | Serious (I²=80% considerable; reduced but still notable) | No serious concerns | No serious concerns | None detected | Low |
| Major Bleeding | Moderate (some risk observed but generally low) | Not serious (I²=38% moderate heterogeneity) | No serious concerns | No serious concerns | None detected | High |
| Myocardial Infarction | Moderate | Not serious (I²=27% moderate) | No serious concerns | No serious concerns | None detected | Moderate |
| Stent Thrombosis | Moderate | Not serious (I²=27% moderate) | No serious concerns | No serious concerns | None detected | Moderate |
| Stroke | Moderate | Not serious (I²=36% moderate) | No serious concerns | No serious concerns | None detected | Low |
| Target Vessel Revascularization | Moderate | Not serious (I²=0% low heterogeneity) | No serious concerns | No serious concerns | None detected | Moderate |
